# Changes in passively‐sensitized basophil activation to αS1‐casein after oral immunotherapy

**DOI:** 10.1002/iid3.294

**Published:** 2020-03-03

**Authors:** Teruaki Matsui, Michihiro Naito, Kazunori Tagami, Iwao Tajima, Miyuki Teshigawara, Atsushi Makino, Katsumasa Kitamura, Yoshihiro Takasato, Shiro Sugiura, Chikako Yamada, Hidehiko Izumi, Ikuya Tsuge, Yasuto Kondo, Komei Ito

**Affiliations:** ^1^ Department of Allergy Aichi Children's Health and Medical Center; ^2^ Department of Nutritional Sciences Nagoya University of Arts and Sciences; ^3^ Department of Nutritional Sciences Kasugai Municipal Hospital; ^4^ Department of Pediatrics Toyohashi Municipal Hospital; ^5^ Department of Pediatrics Fujita Health University

**Keywords:** basophil, immunoglobulin E, immunoglobulin G, immunotherapy, αS1‐casein

## Abstract

**Introduction:**

Immune response to cow's milk allergen (CMA) has been analyzed mostly using crude milk antigen or a mixture of various caseins. This study aimed to assess the changes in the immunological response against αS1‐casein during oral immunotherapy (OIT) and to investigate the mechanism of tolerance.

**Methods:**

We have performed rush OIT to 39 patients with CMA and obtained the serum samples up to 3 years after OIT. Immunoglobulin E (IgE) and IgG4 antibodies specific to highly purified αS1‐casein as well as passively‐sensitized basophil activation were evaluated using the serial samples. Furthermore, we examined whether basophil activation led by the pre‐OIT serum was suppressed by the post‐OIT serum, or by the tolerant serum obtained from naturally outgrown patients.

**Results:**

Specific IgE to αS1‐casein was significantly reduced after OIT. Specific IgG4 (sIgG4) to αS1‐casein was also detected in most of the pre‐OIT sera, which was not significantly increased after OIT. Activation of passively‐sensitized basophils to αS1‐casein was significantly reduced after 2 years (14% ± 19%) and 3 years (19% ± 18%) post‐OIT compared with pre‐OIT (%CD63^high^ basophils; 51% ± 27%). Furthermore, the addition of post‐OIT or tolerant serum to pre‐OIT serum significantly suppressed the basophil activation. This suppression was abrogated by washing the supernatant after passive sensitization, but not by depleting IgG antibodies from post‐OIT or tolerant sera, nor by blocking FcγRIIb using an anti‐FcγR antibody.

**Conclusions:**

αS1‐casein‐sIgG4 plays a minor role in tolerance mechanisms in cases of CMA; humoral factors other than antigen‐sIgG4 may be involved.

## Graphical abstract

Cow's milk oral immunotherapy (OIT) led to an increased tolerated amount of cow's milk. While αS1‐casein‐specific immunoglobulin E decreased and basophil activation stimulated by αS1‐casein was reduced, αS1‐casein‐specific IgG4 did not increase. Antigen‐specific humoral factors other than IgG4 both in post‐OIT and sera of patients with the natural outgrowth of cow's milk allergy could inhibit basophil activation sensitized by pre‐OIT sera.

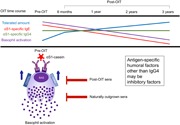

## 1 INTRODUCTION

Cow's milk is a major food allergen for children worldwide^1^; some individuals are able to acquire natural tolerance, whereas others cannot.^1^ The effect of oral immunotherapy (OIT) has been reported in the patients with immunoglobulin E (IgE)‐mediated cow's milk allergy (CMA).^2^, ^3^ OIT help some patients to achieve sustained unresponsiveness; however, others can only acquire temporary desensitization or even a small increase of the threshold dose.^3^, ^4^ The estimated factors involved in the mechanism of desensitization include decreased allergen‐specific IgE (sIgE) and increased specific IgG4 (sIgG4), induction of regulatory T cells, and suppression of mast cells/basophils activation. Nonetheless, the overall perspective of the immunological mechanisms of OIT remain uncertain.^4^, ^5^

Casein, α‐lactalbumin, and β‐lactoglobulin are major allergens in CM.^6^, ^7^ Casein are further fractionated into αS1‐, αS2‐, β‐, and κ‐casein, and all of them have been identified as allergen components.^6^ Among these, αS1‐casein is suggested to have the strongest allergenic activity, as it is resistant to heat denaturation due to its lack of solid three‐dimensional structure and has many sequential IgE epitopes.^8^ However, each casein component should have independent allergenic activities, because the amino acid sequence varies between casein fractions.^8^, ^9^

Although immunological responses could be different among each component, to date, immunological investigations on CMA have predominantly focused on crude milk antigen.^10^, ^11^, ^12^, ^13^, ^14^ Although some studies have investigated casein‐specific immunological changes,^15^, ^16^, ^17^, ^18^, ^19^ analysis of isolated casein fraction‐specific immunological mechanisms, particularly about αS1‐casein have been insufficient.

This study focused on how immunological responses toward single allergen component change during OIT and investigated the mechanism of tolerance. We purified αS1‐casein and investigated immunological changes in response to OIT for the patients with CMA. We employed a passively‐sensitized (PS‐) basophil activation test to investigate the humoral factors which suppressed the allergic reaction after OIT.

## 2 METHODS

This study was approved by the Research Ethics Board of Aichi Children's Health and Medical Center (approval number: 201669). The study was conducted in accordance with the principles embodied in the Declaration of Helsinki (1965). Written informed consent was obtained from all caregivers, and patient anonymity was preserved using methods approved by the ethics committee.

### 2.1 OIT protocol

We recruited the patients with CMA who were unexpected to acquire natural tolerance. The inclusion criteria of CM‐OIT were patients aged ≥5 years who had a threshold dose of ≤5 mL of CM as determined by an open oral food challenge (OFC) test.

The OIT protocol consisted of a rush phase for 12 days of hospitalization, followed by a slow increase and maintenance phases. Each patient underwent an OFC within 6 months before OIT, and the initial treatment dose was determined based on the threshold dose and the severity of induced symptoms. In the rush phase, patients consumed commercially available pasteurized CM as often as four times per day, increasing each dose by approximately 1.3 times unless a severe symptom was evoked. After the rush phase, patients continued to have the maximum tolerated amount at their discharge once daily for several months. If severe or frequent symptoms were not observed, they slowly increased the dose up to 200 mL of CM (slow‐increase phase) and kept the programmed intake (maintenance phase). During the rush phase, 37 patients (97.4%) experienced any symptoms, and 34 (87.2%) had to use medications to relieve symptoms. One patient (2.6%) needed intramuscular adrenaline injection. In total, 880 ingestions were tried for all participants during the rush phase. Among them, any symptoms occurred in 356 (40.5%), any medications were needed in 140 (15.9%), and intramuscular adrenaline injection was needed in 2 (0.2%).

### 2.2 Target serum

OIT was conducted in 39 participants from April 2011 to March 2017. Twenty‐five were males, and the median age at the initiation of OIT was 8 years (interquartile range [IQR], 6‐10 years). As a clinical history, 82% experienced CM‐induced anaphylaxis, 90% had atopic dermatitis and 62% had bronchial asthma. The median threshold dose at pre‐OIT determined by the OFC was 2.0 mL (IQR, 2.0‐5.0 mL). The sera were collected at pre‐OIT and 6 months, 1, 2, or 3 years after OIT (post‐OIT). Furthermore, we employed seven “tolerant sera” from patients with naturally outgrown CMA.

### 2.3 Purification of αS1‐casein

The αS1‐casein fraction was purified from pasteurized CM based on a method reported by Igarashi et al^20^ with some modifications. CM was mixed with ethanol, 4M NaSCN, and 0.75M CaCl_2_ and centrifuged at 9800*g* for 30 minutes. The precipitate was dissolved in 4M urea containing 0.04M NaCl and 0.03M EDTA. Then, 1% 2‐mercaptoethanol, 0.2M Na_2_HPO_4_, and 2M CaCl_2_ were added to the suspension and centrifuged at 1900*g* for 10 minutes. The precipitate was dissolved in 4M urea, 0.03M EDTA, and 3.2M (NH_4_)_2_SO_4_ and centrifuged. The resulting precipitate was resuspended in water and an equal volume of ethanol and centrifuged. Proteins in the supernatant were precipitated by adjusting its pH to 4.7 using HCl and dissolved in 4M urea and 0.1M NH_4_H_2_PO_4_ and centrifuged. The precipitate containing αS1‐casein was separated by size‐exclusion high‐performance liquid chromatography.

### 2.4 Sodium dodecyl sulfate‐polyacrylamide gel electrophoresis and immunoblotting

The purity of the isolated αS1‐casein was confirmed by sodium dodecyl sulfate‐polyacrylamide gel electrophoresis and immunoblotting using 15% polyacrylamide gels, according to the methods by Laemmli^21^ and Towbin et al^22^ (Figure S1). The gels were stained with Coomassie Brilliant Blue R‐250. For immunoblotting, proteins were transferred to polyvinylidene difluoride membranes. After blocking, membranes were incubated with anti‐α‐casein mouse monoclonal antibody diluted 1:2000 (Cosmo Bio, Tokyo, Japan). Peroxidase‐conjugated anti‐mouse IgG diluted 1:5000 (Jackson ImmunoResearch, PA) was served as the secondary antibody.

### 2.5 Enzyme‐linked immunosorbent assay for αS1‐casein‐specific immunoglobulin

sIgE to CM, casein, α‐lactalbumin, and β‐lactoglobulin were detected using ImmunoCAP (Thermo Fisher Diagnostics, Tokyo, Japan).

The levels of sIgE and sIgG4 to αS1‐casein were measured using in‐house enzyme‐linked immunosorbent assay (ELISA).^23^ The 96‐well ELISA plates (Nunc) were coated with 10 µg/mL αS1‐casein in phosphate‐buffered saline (PBS). After washing, the plates were blocked with protein‐free blocking buffer (Thermo Fisher Scientific) containing 0.05% Tween 20 (PFBBT) at 4°C for 8 hours. The serum samples were diluted 1:10 (IgE) or 1:200 (IgG4) in PFBBT and incubated overnight at 4°C. After washing, AP‐conjugated goat anti‐human IgE (diluted 1:1000; Bethyl Laboratories) or horseradish peroxidase‐conjugated mouse anti‐human IgG4 (diluted 1:10 000; Thermo Fisher Scientific) was added. All assays were concurrently performed in duplicate. The relative immunoglobulin levels (%) for each sample were calculated based on the reference serum with high αS1‐CN‐specific immunoglobulin levels, which was set at 100%.

### 2.6 Passive sensitization of IgE‐stripped basophils

Cell surface IgEs were stripped from the basophils collected from a healthy adult donor without CMA.^24^, ^25^ First, 13 mL of HA buffer (0.3% human serum albumin, 10 mM HEPES, 140 mM sodium chloride, and 5 mM potassium chloride adjusted to pH 7.4 at 4°C) was added to 2 mL of whole blood. After centrifugation at 1500 rpm for 7 minutes, the supernatant was discarded, and 13 mL of lactic acid buffer (28 mM lactic acid, 140 mM sodium chloride, and 5 mM potassium chloride adjusted to pH 3.9 at 4°C) was added. The cells were incubated on ice for 5 minutes for IgE stripping, then washed twice using HA buffer. We confirmed the IgE stripping by FACS analysis detecting a decrease in surface IgE and an increase in CRA2, which is the binding site of IgE on the FcεRIα (Figure S2).

For passive sensitization, equal volumes of the target serum and PBS were added to the IgE‐stripped basophils and incubated at 37°C for 1 hour, then overnight at 4°C.

### 2.7 PS‐basophil activation test

A PS‐basophil activation test was performed using the Allergenicity kit (Beckman Coulter, Brea, CA) along with the anti‐CD63 antibody (Anti‐Hu CD63‐APC; EXBIO, Praha, Vestec, Czech Republic). First, 50 μL of activation buffer, 15 μL of antibody set (CD3‐PC7/CRTH2‐FITC/CD203c‐PE and CD63‐APC at a ratio of 2:1), and 10 µL of antigen solution were added to 50 μL of PS‐basophils and incubated for 15 minutes at 37°C. The antigen solutions were either αS1‐casein (1 mg/mL), an anti‐IgE antibody for the positive control, or PBS for the negative control. The reaction was stopped by adding 50 μL of the stop solution, then 1 mL of fix and lyse solution was added. After washing with PBS and centrifuging at 4000 rpm for 5 minutes, cells were resuspended in 0.5 mL PBS containing 0.1% formaldehyde. Cell‐surface CD3, CRTH2, CD203c, and CD63 were detected using a flow cytometer (Gallios; Beckman Coulter) and analyzed using Kaluza software (Beckman Coulter). Basophils were identified in the leukocytes, gated using front and side scatter, as CD3^−^ CRTH2^+^ cells (Figure S3). Activated basophils were detected as the percentage of either CD203c^high^ or CD63^high^ basophils, which were gated over the top 5% of the expression of negative controls (Figure S3). The change of mean fluorescence intensity (MFI) of CD203c or CD63 was also analyzed as the marker of basophil activation.

### 2.8 Evaluation of cell surface antigen

We aimed to confirm that purified αS1‐casein was bound to cell surface sIgE and activated the PS‐basophils. Purified αS1‐casein was labeled with biotin using the Biotin Labeling Kit‐NH2 (Dojindo Molecular Technologies, Kumamoto, Japan). Biotin‐labeled αS1‐casein was added to PS‐basophil suspension and incubated on ice for 30 minutes. After washing, anti‐human IgE‐PE (BioLegend, San Diego, CA), CRA1 (anti‐human FcεRIα‐PE/Cy7, detecting non‐IgE‐binding site on FcεRIα; BioLegend), CRA2 (anti‐human FcεRIα‐FITC, detecting IgE‐binding site on FcεRIα; BioAcademia, Osaka, Japan), anti‐human CD3‐APC/Alexa Fluor 750 (Beckman Coulter), anti‐human CRTH2‐PerCP/Cy5.5 (BioLegend), and streptavidin‐Brilliant violet 421 (BioLegend) were added. The reaction was allowed to proceed for 30 minutes in the dark at 4°C and was stopped by adding the stop solution. After adding Fix and Lyse solution and washing, cells were resuspended in 0.5 mL PBS containing 0.1% formaldehyde and analyzed using the flow cytometer.

### 2.9 Suppressive effect of post‐OIT sera on the PS‐basophil activation

Post‐OIT or tolerant sera were added to the pre‐OIT sera, instead of PBS, during passive sensitization in some experiments to examine the inhibitory activities of these sera.

To assess whether IgG was correlated to the suppression of basophil activation, IgG antibody in post‐OIT or tolerant sera was removed using the Nab Protein G Spin Kit (Thermo Fisher Scientific) before passive sensitization according to the manufacturer's manual. To analyze whether inhibitory signals from FcγRII (CD32) contributed to the suppression of basophil activation, anti‐human CD32 (2 µg/mL, FUN2; BioLegend) was added during passive sensitization according to the methods used by Burton et al.^26^ We matched the same individual's pre‐OIT and post‐OIT sera for the experiments. The backgrounds of the post‐OIT sera used for these experiments are listed in Table S1.

### 2.10 Statistical analyses

One‐way analysis of variance (ANOVA) test was used to analyze continuous variables, and the Dunnett's test was applied as a post hoc analysis. Pearson's product–moment correlation coefficient was used to test correlations between continuous variables. Generalized estimating equations analysis, which is a marginal model applied for longitudinal data analysis, was applied additionally to analyze the changes of αS1‐casein‐sIgG4. *P* value less than .05 was considered statistically significant. Statistical analyses were performed using the EZR software package (Saitama Medical Center, Jichi Medical University, Saitama, Japan)^27^ or STATA software program (version 12.1 for MAC; STATA Inc, College Station, TX).

## 3 RESULTS

### 3.1 Clinical outcome and change in immunoglobulin levels associated with OIT

Compared with pre‐OIT, the median (IQR) tolerated amount of CM significantly increased to 100 mL (40‐100) at 6 months, 110 mL (62‐170) at 1 year, 200 mL (60‐200) at 2 years, and 190 mL (91‐200) at 3 years post‐OIT (Table 1).

**Table 1:** Changes in parameters in response to oral immunotherapy

	Pre	6 mo	1 y	2 y	3 y
Tolerated amount, mL	0 (0‐0.2)	100 (40‐100)**	110 (62‐170)**	200 (60‐200)**	190 (91‐200)**
Total IgE, IU/mL	710 (340‐1500)	580 (290‐1200)	760 (300‐1800)	660 (380‐1900)	1700 (780‐2700)
Cow's milk‐specific IgE, kU_A_/L	22 (12‐56)	11 (5.9‐27)*	10 (5.0‐31)	3.7 (2.6‐8.5)**	7.8 (2.9‐22)
α‐Lactalbumin‐specific IgE, kU_A_/L	2.4 (0.34‐12)	1.3 (0.34‐4.4)	1.1 (0.34‐7.1)	0.35 (0.34‐0.93)	0.64 (0.34‐4.6)
β‐Lactoglobulin‐specific IgE, kU_A_/L	1.0 (0.34‐6.2)	0.93 (0.34‐3.4)	0.81 (0.34‐3.0)	0.46 (0.34‐1.5)	0.34 (0.34‐0.95)
Casein‐specific IgE, kU_A_/L	25 (13‐47)	11 (5.9‐26)*	10 (5.0‐31)*	4.2 (2.4‐10)**	8.5 (2.7‐20)*
αS1‐casein‐specific IgE (%)	30 (22‐52)	20 (11‐33)	18 (8.5‐27)*	9.4 (5.8‐15)**	9.6 (8.0‐26)*
αS1‐casein‐specific IgG4 (%)	12 (4.9‐35)	20 (5.9‐46)	28 (6.1‐83)	32 (15‐71)	37 (3.5‐56)
αS1‐casein‐specific IgG4/IgE	0.44 (0.23‐0.91)	0.85 (0.49‐1.4)	1.4 (0.68‐2.5)**	3.2 (1.8‐4.3)**	1.4 (0.42‐4.0)

*Note*: Median values and interquartile range are shown in parentheses. Parameters at 6 mo, 1 y, 2 y, 3 y were compared with pre‐OIT using the Dunnett's test (n = 39).

Abbreviations: IgE, immunoglobulin E; IgG, immunoglobulin G; OIT, oral immunotherapy.

* *P* < .05.

** *P* < .01.

sIgE levels to CM, casein, and αS1‐casein were significantly reduced after OIT. sIgG4 to αS1‐casein was not significantly increased, because relatively high sIgG4 levels were detected at pre‐OIT in most of the patients (Table 1).

In the analysis of all data points, the tolerated amount of CM was inversely correlated to sIgE levels to CM (*r* = −.42), casein (*r* = −.44), and αS1‐casein (*r* = −.46) (Table S2). However, no correlation was observed between the tolerated CM amount and sIgG4 levels to αS1‐casein (*r* = .078). Due to the decrease in sIgE levels after OIT, the αS1‐casein‐sIgG4/IgE ratio was found to be correlated to the tolerated amount of CM (*r* = .42). However, it was not superior to that of sIgE alone Tables 1 (*r* = −.46; Table S2).

### 3.2 PS‐basophil activation

**Figure 1:**
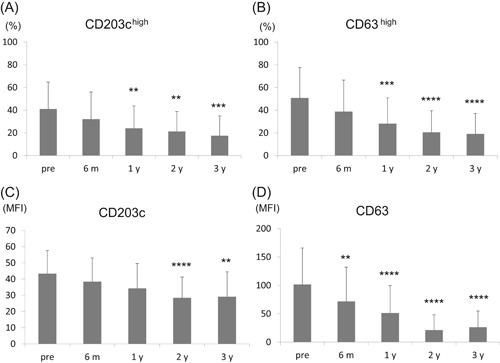
Changes in basophil activation stimulated by αS1‐casein by oral immunotherapy, basophils from a healthy nonmilk allergy donor were passively sensitized using participants’ sera before oral immunotherapy (OIT); pre‐OIT (n = 39) and post‐OIT (6 months, 6 m [n = 36]; 1 year, 1 y [n = 28]; 2 years, 2 y [n = 16]; 3 years after OIT; 3 y [n = 14]), then stimulated by αS1‐casein. Basophil activation was analyzed using the percentages of high expression and mean fluorescence intensity (MFI) of CD203c (A and C) and CD63 (B and D). Parameters at 6 m, 1 y, 2 y, and 3 y were compared with pre‐OIT parameters using the Dunnett's test (n = 39). **P < .01, ***P < .005, ****P < .001

Pre‐OIT sera induced activation of PS‐basophils in response to αS1‐casein as 41 ± 23% of CD203c^high^ and 51 ± 27% of CD63^high^ basophils. The percentage of activated basophils was significantly decreased at 1‐year post‐OIT and later (Figure 1A,B). The MFI of CD203c‐FITC and CD63‐PE also decreased time‐dependently after OIT (Figure 1C,D).

**Figure 2:**
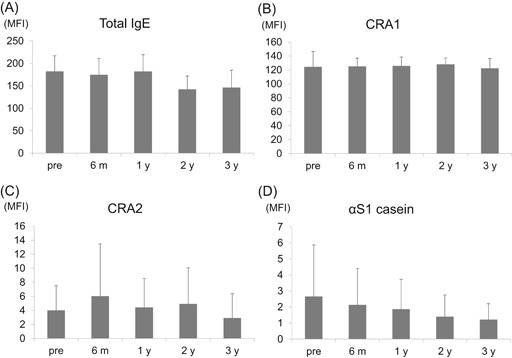
Changes in basophil surface antigens stimulated by αS1‐casein by oral immunotherapy, basophils from a healthy nonmilk allergy donor were passively sensitized using participants’ sera at the beginning of the oral immunotherapy (OIT); pre‐OIT (n = 39), and post‐OIT (6 months, 6 m [n = 36]; 1 year, 1 y [n = 28]; 2 years, 2 y [n = 16]; 3 years after OIT; 3 y [n = 14]), then stimulated by αS1‐casein. Cell surface markers were detected by FACS analysis. (A) Total IgE, (B) CRA1, (C) CRA2, and (D) αS1‐casein. One‐way analysis of variance test revealed no statistically significant difference (n = 39). IgE, immunoglobulin E

Analysis of cell surface markers revealed that total IgE (Figure 2A), CRA1 (nonbinding site of IgEs on FcεRIα; Figure 2B), and CRA2 (binding site of IgEs on FcεRIα; Figure 2C) remained unchanged. Cell surface αS1‐casein estimated to be bound to cell surface sIgE or sIgG antibodies, slightly reduced over time, although this did not reach statistical significance (Figure 2D).

In the analysis of all data points, the αS1‐casein‐sIgE level was correlated to both the percentage of activated basophils (CD63^high^: *r* = .54; CD203c^high^: *r* = .48) and the MFI the activation markers (CD63^high^: *r* = .65; CD203c^high^: *r* = .58) (Table S3). However, a negative correlation was not observed between the αS1‐casein‐sIgG4 level and basophil activation. Moreover, when we only focused on the data at the pre‐OIT, the sIgG4 level was not correlated to PS‐basophil activation (CD63^high^: *r* = .30, *P* = .066; CD203c^high^: *r* = .25, *P* = .13).

A marginal negative correlation was observed between IgG4/IgE ratio and PS‐basophil activation, which was commonly due to the decrease in sIgE levels (Table S3).

The tolerated amount of CM at every data point inversely correlated with the percentage of activated basophils and the MFI of the activation markers (Table S4).

### 3.3 Humoral factors contributing to the reduction of activated basophils

To examine the presence of inhibitory factors in post‐OIT and tolerant sera, post‐OIT or tolerant sera were mixed with pre‐OIT sera at the passive sensitization of basophils.

**Figure 3:**
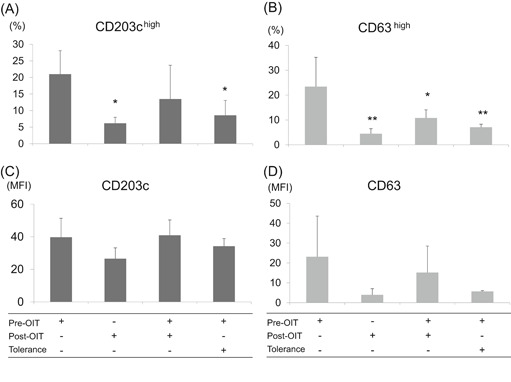
Suppression of the basophil activation stimulated by αS1‐casein, Basophils from a healthy nonmilk allergy donor were passively sensitized using participants’ sera at the beginning of the oral immunotherapy (OIT) (pre‐OIT) plus PBS, post‐OIT plus PBS, pre‐OIT plus post‐OIT, or pre‐OIT plus naturally outgrown from milk allergy (tolerance), then stimulated by αS1‐casein. Basophil activation was analyzed using the percentages of high expression and mean fluorescence intensity (MFI) of CD203c (A and C) and CD63 (B and D). Parameters of post‐OIT plus PBS, pre‐OIT plus post‐OIT, and pre‐OIT plus tolerance were compared with parameters of pre‐OIT plus PBS using the Dunnett's test (n = 4). *P < .05, **P < .01

Supplementation of post‐OIT or tolerant sera significantly suppressed the percentage of CD63^high^ basophils, and the latter suppressed the percentage of CD203c^high^ basophils stimulated with αS1‐casein (Figure 3). In contrast, both sera did not suppress the basophil activation caused by anti‐IgE stimulation (Figure S4).

**Figure 4:**
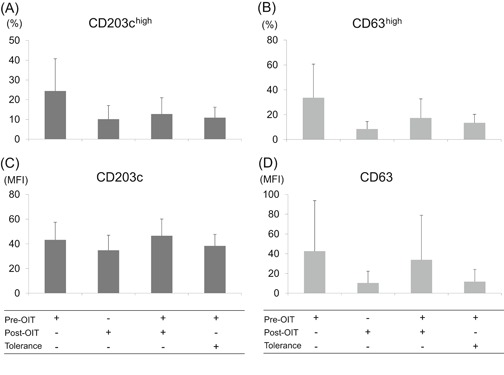
Suppression of basophil activation stimulated by αS1‐casein after washing the supernatant, basophils from a healthy nonmilk allergy donor were passively sensitized using participants’ sera at the beginning of the oral immunotherapy (OIT) (pre‐OIT) plus PBS, post‐OIT plus PBS, pre‐OIT plus post‐OIT, or pre‐OIT plus sera of patients with natural outgrowth of cow's milk allergy (tolerance) as described in Figure 3, washing the supernatant using PBS, then stimulating with αS1‐casein. Basophil activation was analyzed using the percentages of high expression and mean fluorescence intensity (MFI) of CD203c (A and C) and CD63 (B and D). One‐way analysis of variance test revealed no statistically significant difference (n = 4)

To ascertain the mechanism of this suppression, we washed out the supernatant of the PS‐basophils before stimulation with αS1‐casein. As a result, inhibition of the basophil activation partially remained, but the statistical significance was disappeared (Figure 4).

We then investigated the role of IgG4 antibody in the post‐OIT or tolerant sera by depletion of IgG antibodies from these sera. The depletion of IgG from post‐OIT or tolerant sera did not abolish the suppressive effect on the basophil activation (Figure S5). Moreover, the suppressive effect of post‐OIT and tolerant sera was not abolished by blocking of FcγRIIb (Figure S6).

## 4 DISCUSSION

The present study analyzed the immunological changes during CM‐OIT using the PS‐basophil activation tests stimulated with αS1‐casein, an isolated CM allergen component.

Several reports have shown the decrease in basophil activation using fresh blood samples stimulated with crude CM antigen during the course of CM‐OIT.^28^ However, in the present study, the PS‐basophil activation test^25^, ^29^ was used, which facilitated the direct evaluation of the time course of reaction retrospectively and the examination of humoral factors that affect the reaction in the same panel of the experiment.

In accordance with previous studies that used fresh basophils and crude CM antigen, PS‐basophil activation stimulated with αS1‐casein consecutively decreased during the course of OIT.

Both αS1‐casein‐sIgE (Table 1) and PS‐basophil activation were reduced after OIT (Figure 1), and basophil activation was correlated with αS1‐casein‐sIgE (Table S3). Therefore, this raises the possibility that the decrease in PS‐basophil activation might be simply attributed to the decreased amount of sIgE bound on the surface of IgE‐stripped basophils. However, IgEs or αS1‐casein on basophils of post‐OIT did not significantly decrease compared with pre‐OIT (Figures 2A and 2D). Moreover, the admixture of post‐OIT sera with pre‐OIT sera suppressed the basophil activation compared with the admixture of the pre‐OIT with PBS even though sIgEs were more abundant in the former admixture (Figure 3).

These results suggest that some humoral factors rather than reduced IgE are related to the suppressive mechanisms of basophil activation.

We further confirmed that the humoral factors do exist in the post‐OIT and tolerant sera because supplementation of post‐OIT or tolerant sera during the passive sensitization of IgE‐stripped basophils suppressed PS‐basophil activation caused by the pre‐OIT sera (Figure 3). This suppression may be allergen‐specific because the post‐OIT or tolerant sera did not suppress basophil activation caused by anti‐IgE as the positive control (Figure S4). The humoral factor was present in the post‐OIT sera because the removal of sera after the passive sensitization of basophils partially inhibited the suppressive effect of post‐OIT sera (Figure 4).

Previous reports have indicated that the sIgG4 antibody is the major humoral factor involved in the efficacy of OIT. Moreover, CM‐sIgG4^12^, ^14^, ^30^ and casein‐sIgG4^15^, ^19^ levels increase during the course of CM‐OIT before a decrease in CM‐sIgE is observed. Generally, the IgG4 antibody acts as the neutralizing antibody that occupies the IgE‐binding epitopes in the allergen component.^31^ It also binds to FcγRIIb on the surface of basophils, and the antigen‐dependent cross‐link of FcεRI and FcγRIIb induces inhibitory signal transduction via the activation of immunoreceptor tyrosine‐based inhibition motif.^31^

These mechanisms have been proved in the peanut OIT model. Santos et al^29^ showed that peanut‐induced mast cell activation was inhibited by plasma with detectable peanut‐sIgG4 from peanut‐sensitized but tolerant patients, and this inhibition was partially restored after IgG4 depletion. Burton et al^26^ also reported that post‐OIT sera suppressed basophil activation caused by pre‐OIT sera, and this suppression was blocked by antibodies against FcγRII.

However, the results of the present study were not in accordance with those of previous studies. As per one‐way ANOVA, the αS1‐casein‐specific IgG4 level did not significantly increase by OIT (Table 1), although the generalized estimating equation analysis detected a slight increase. It might be attributed to the high levels of sIgG4 to αS1‐casein detected in several participants before OIT, and the levels did not increase during the course of CM‐OIT. The levels of sIgG4 or IgG4/IgE ratio did not correlate with PS‐basophil activation (Table S3) nor tolerated amount of CM after OIT (Table S2). Moreover, the representative samples showed that neither IgG depletion (Figure S5) nor blocking FcγRIIb (Figure S6) abolished the suppressive effect of post‐OIT and tolerant sera on the basophil activation caused by the pre‐OIT sera.

Recently, Patil et al^32^ have reported similar findings in peanut OIT, where immunoglobulins accounted for only 20.8% of the change in basophil sensitivity to Ara h 2, with Ara h 2‐sIgE levels having the largest contributions, not IgG4 levels. Moreover, they have investigated the suppressive effect of post‐OIT sera on the activation of passively‐sensitized basophils and indicated that neutralizing antibody activity, rather than concentration, might be correlated to clinical reactivity.

Taken together, the present study showed that some antigen‐specific humoral factors in post‐OIT and tolerant sera were involved in the suppression of basophil activation. However, the factor might not simply be the amount of sIgG4 antibodies.

Epitope‐specific antibody binding profiles have been found to be correlated to the development of sustained unresponsiveness after CM‐OIT.^33^ The data clearly showed that CM‐OIT reduced the amount of IgE and increased the amount of IgG4 antibodies specific to the representative epitope. However, the profile of the IgG4 epitope was not parallel to that of the IgE epitope. The best model for the prediction of the likelihood of achieving sustained unresponsiveness that consisted of the baseline profiles of the IgE‐binding epitope alone was developed. Additional models combining IgE and IgG4 epitopes did not improve the performance of the prediction model, and these findings indicated that the role of the sIgG4 antibody on the achievement of desensitization is limited.

The present study had several limitations. First, due to the lack of stocked sera, some post‐OIT data are missing. Second, since a standard value is not available, the measurement of sIgE and sIgG4 levels to αS1‐casein was based on the relative absorbance values of the reference serum. Third, the suppression experiment was conducted with a limited number of representative samples due to the lack of appropriate sera. Fourth, the PS‐basophil activation model does not completely reproduce the immune mechanisms of the patient's fresh basophils, such as the number of FcεRI and FcγRIIb on the surface of the basophils. Finally, the actual association between sIgE and IgG4 antibodies should be examined in an epitope‐specific manner.

In conclusion, our findings of the immunological responses against the single allergen component, αS1‐casein suggested that some antigen‐specific humoral factors might be developed after CM‐OIT, which was involved in the suppression of basophil activation and contributed to the mechanism of tolerance. The presence of the sIgG4 antibody was not sufficient to fully represent the contributing factors, and further investigation to find the additional factor may be expected.

## CONFLICT OF INTERESTS

The authors declare that there are no conflict of interests.

## AUTHOR CONTRIBUTION

TM and KI conceived and designed the study. KT, TM, KK, and YT collected and sorted the stored samples for the study. MN, CY, and HI performed αS1‐casein purification and ELISA. TM, MN, IT, MT, AM, IT, and YK performed passive sensitization of the basophils and basophil activation test. TM, MT, IT, YK, SS, and KI performed the analysis of the experimental data and statistical analysis. The manuscript was written by TM, MN, and KI in collaboration with all the contributing authors.

## Supporting information

Supporting information

Supporting information

## Data Availability

The data that support the findings of this study are available on request from the corresponding author. The data are not publicly available due to privacy or ethical restrictions.
