# Supplementary material for: Changes in passively‐sensitized basophil activation to αS1‐casein after oral immunotherapy
Source: Immun Inflamm Dis. 2020 Mar 3;8(2):188–97. doi: 10.1002/iid3.294 (PMC7212200; doi:10.1002/iid3.294)
Supplement: Supplementary file 1 — Supporting information [file IID3-8-188-s001.pdf]

Supplementary Figure 1. Purification of αS1-casein

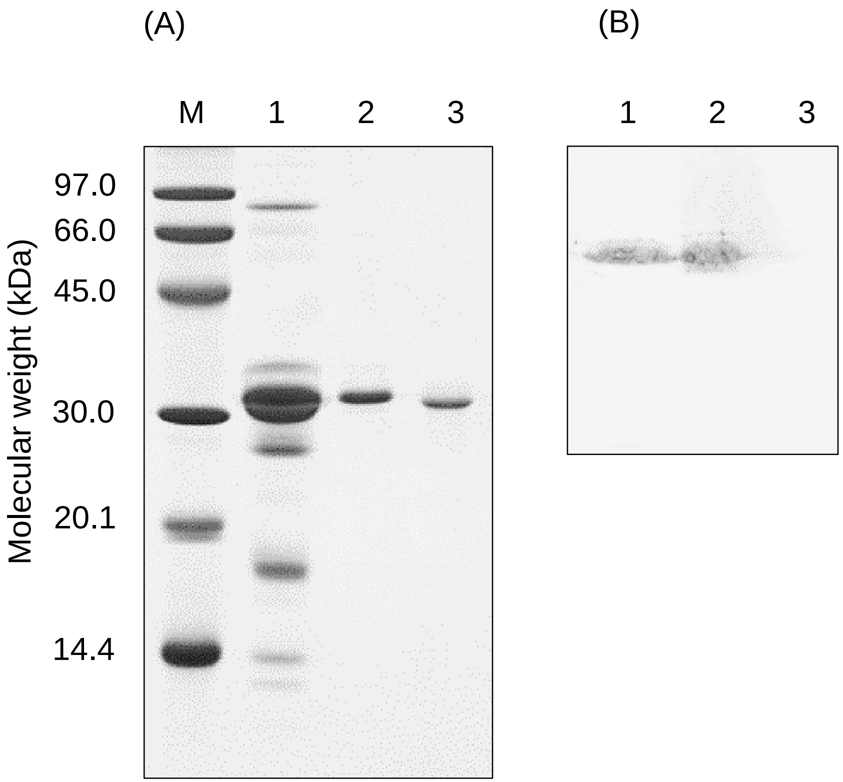

αS1-casein was purified from cow's milk and confirmed using sodium dodecyl sulfate-polyacrylamide gel electrophoresis (A) and immunoblotting (B).

Anti-α-casein mouse monoclonal antibody was used for immunoblotting.

Lane M, molecular weight marker; Lane 1, cow's milk; Lane 2, αS1-casein; Lane 3, β-casein.

Supplementary Figure 2. Confirmation of IgE stripping from basophils

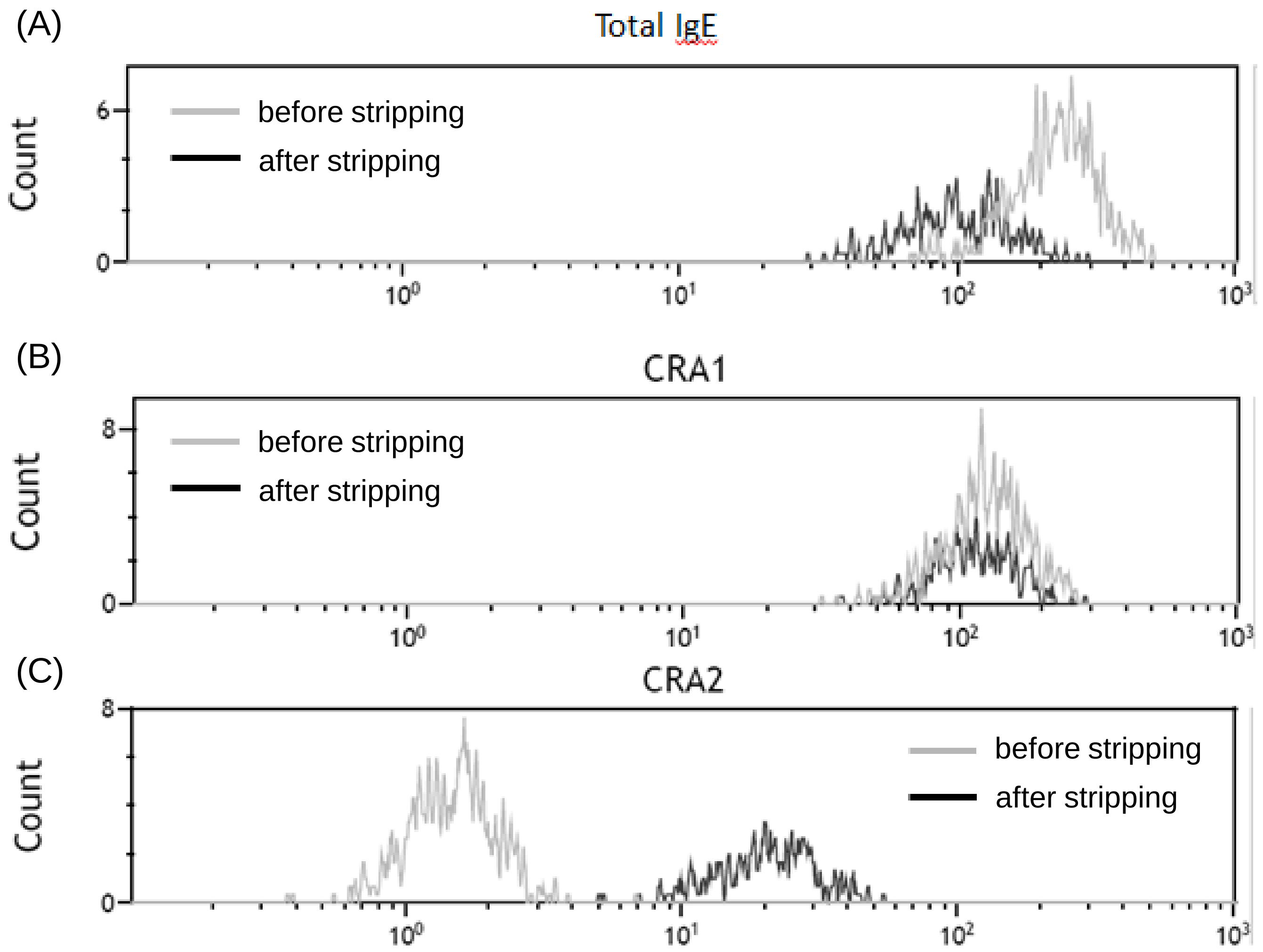

IgEs were stripped from healthy donor basophils by incubation with lactic acid buffer and stripping was confirmed by decreased total IgE levels and increased CRA2 levels; CRA2 is the binding site of IgEs on FcεRIa. Gray lines represent cells before stripping, and dark lines represent cells after stripping.

Supplementary Figure 3. Gating strategy of basophils and activated basophils

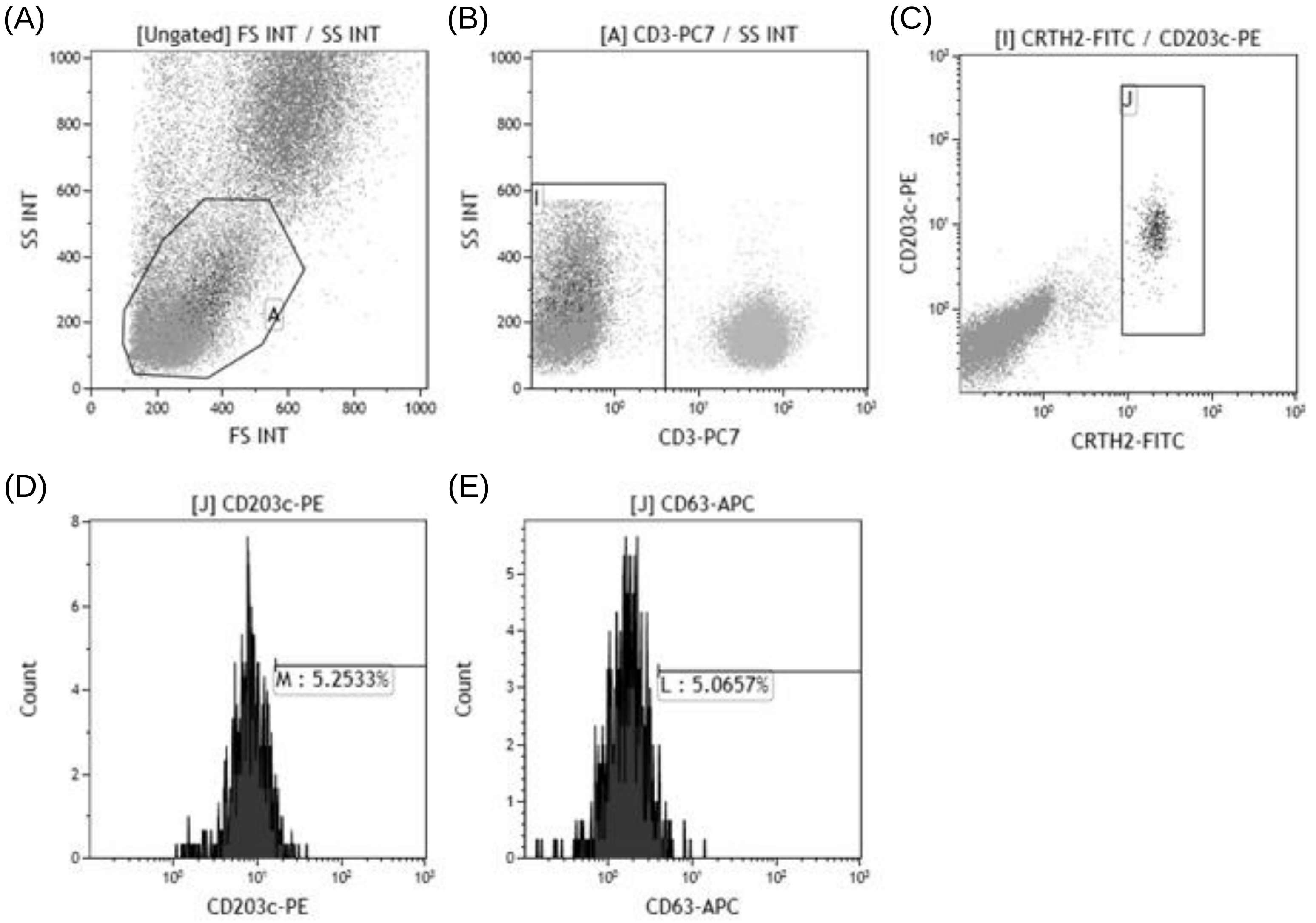

Leukocytes were gated by front scatter and side scatter (A). T lymphocytes were screened by the property of CD3 positivity (B). Basophils were gated using CRTH2-positive cells (C). Activated basophils were defined as the cells in the top 5% gate of CD203c (D) and CD63 (E) expression in the non-stimulated controls.

Supplementary Figure 4. Suppression of basophil activation stimulated by anti-IgE antibody

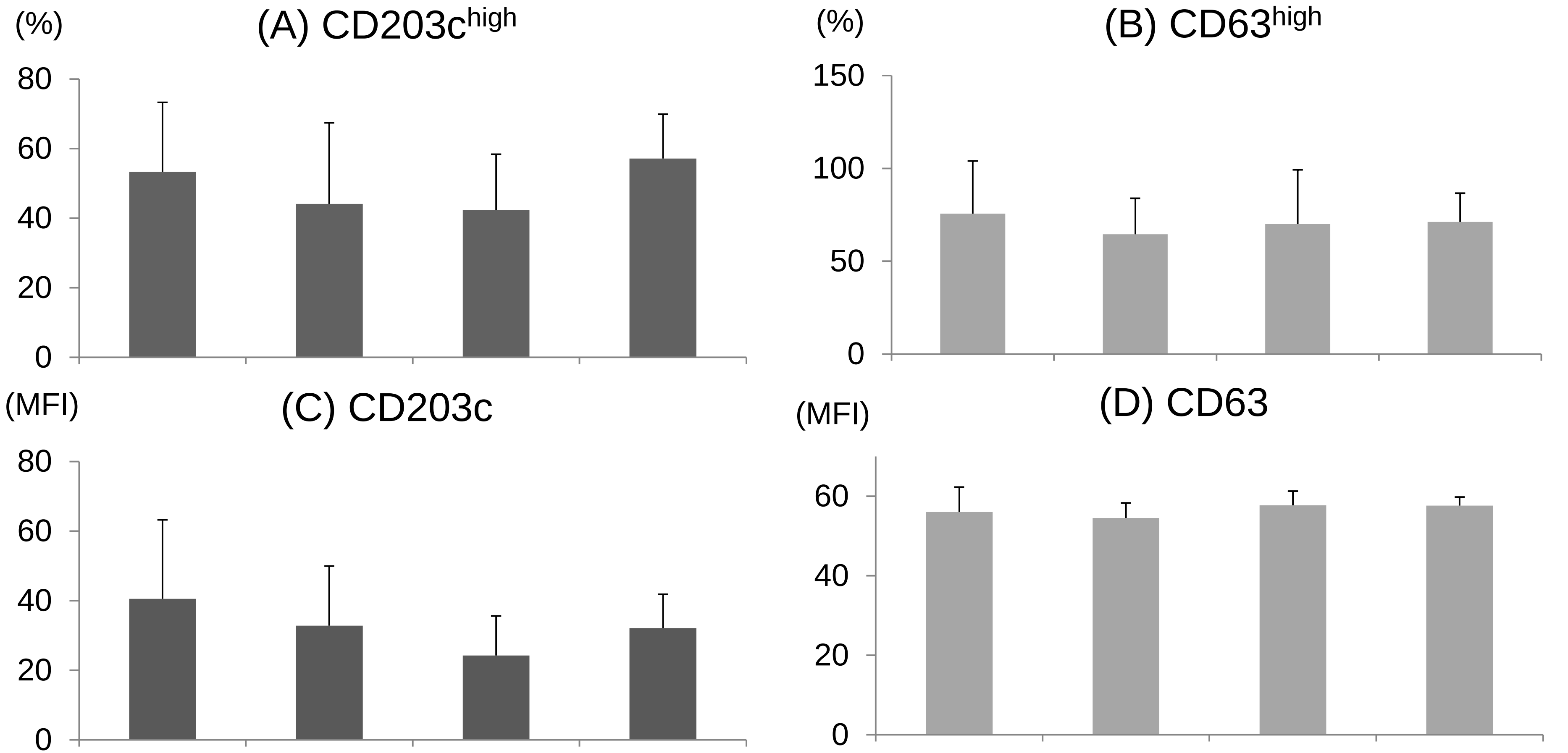

Pre-OIT	+	−	+	+
Post-OIT	−	+	+	−
Tolerance	−	−	−	+

Pre-OIT	+	−	+	+
Post-OIT	−	+	+	−
Tolerance	−	−	−	+

Basophils from a healthy nonmilk allergy donor were passively sensitized using participants' sera at the beginning of the oral immunotherapy (OIT) (pre-OIT) plus PBS, post-OIT plus PBS, pre-OIT plus post-OIT, or pre-OIT plus sera of patients with natural outgrowth of cow's milk allergy (tolerance) and then stimulated using anti-IgE antibodies. Basophil activation was analyzed using the percentages of high expression and mean fluorescence intensity (MFI) of CD203c [(A) and (C)] and CD63 [(B) and (D)]. One-way ANOVA revealed no statistically significant difference (n = 4).

Supplementary Figure 5. Effect of IgG depletion in the Post-OIT and Tolerance sera

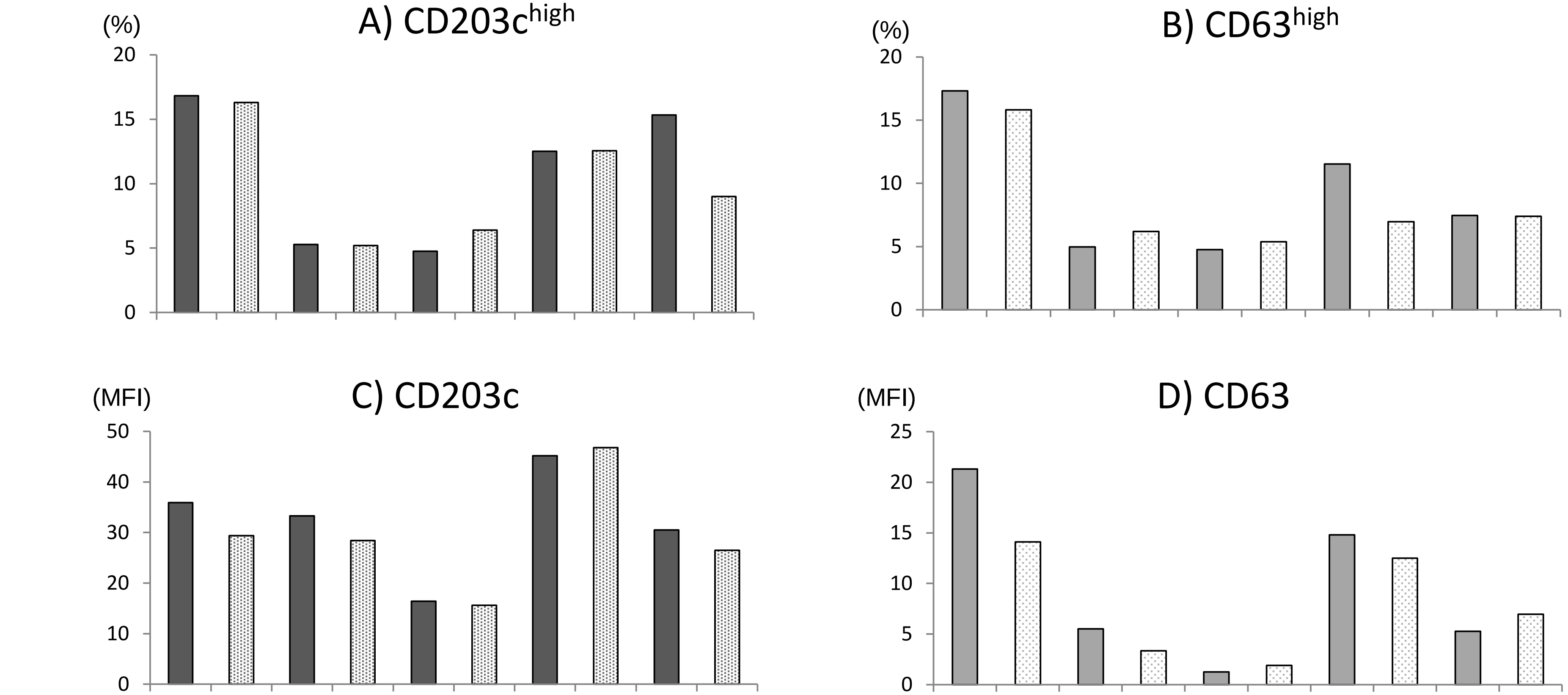

Pre-OIT	+	+	-	-	-	-	+	+	+	+
Post-OIT	-	-	+	+	-	-	+	+	-	-
Tolerance	-	-	-	-	+	+	-	-	+	+
IgG depletion	-	+	-	+	-	+	-	+	-	+

Pre-OIT	+	+	-	-	-	-	+	+	+	+
Post-OIT	-	-	+	+	-	-	+	+	-	-
Tolerance	-	-	-	-	+	+	-	-	+	+
IgG depletion	-	+	-	+	-	+	-	+	-	+

Basophils from a healthy nonmilk allergy donor was passively sensitized using one of the participants' sera at the beginning of the oral immunotherapy (OIT) (pre-OIT), post-OIT, natural outgrowth of cow's milk allergy (tolerance) with or without IgG depletion by protein G column, then stimulated by αS1-casein. Basophil activation was analyzed using the percentages of high expression and mean fluorescence intensity (MFI) of CD203c [(A) and (C)], and CD63 [(B) and (D)]. This is the representative data from the 2 experiments.

Supplementary Figure 6. Effect of anti-CD32 antibody in the Post-OIT and Tolerance sera

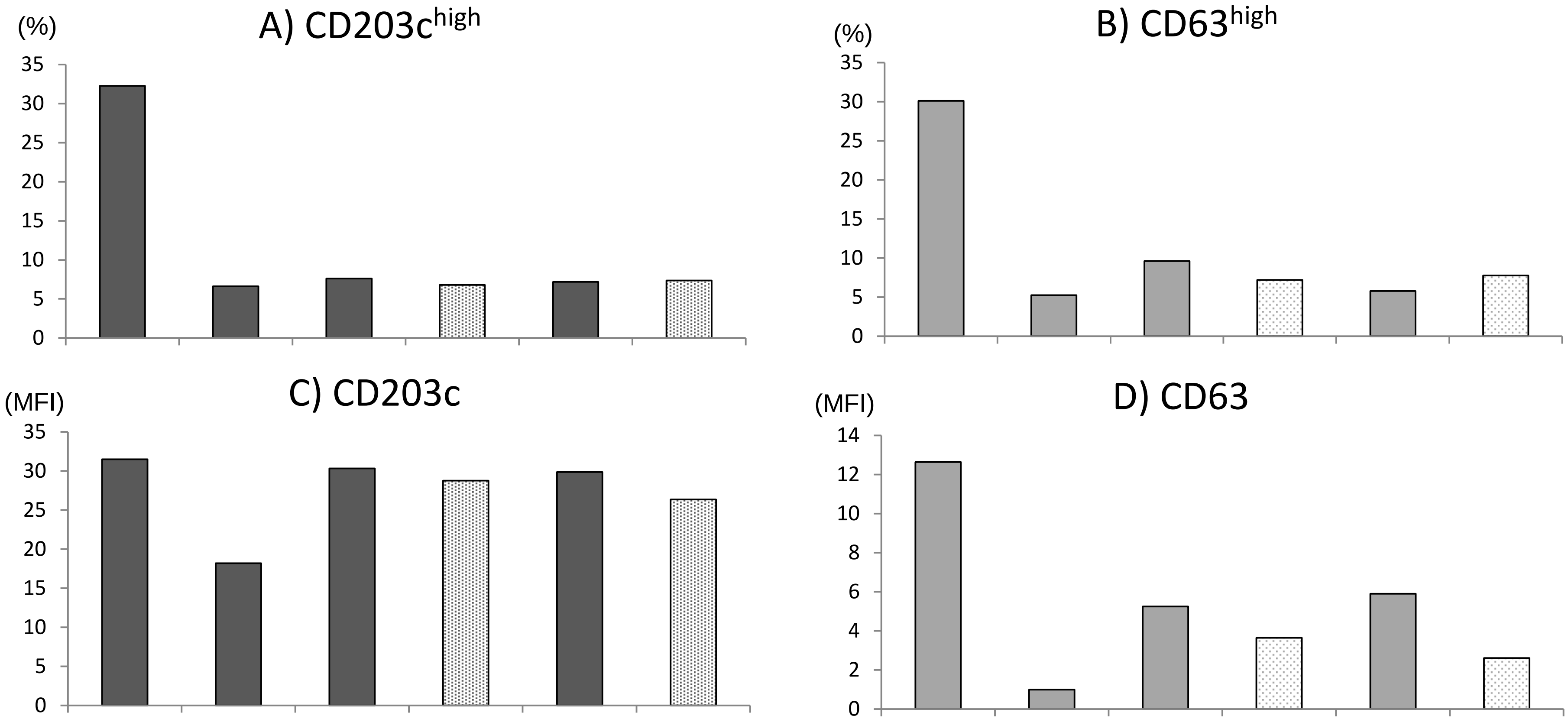

Pre-OIT	+	-	+	+	+	+
Post-OIT	-	+	+	+	-	-
Tolerance	-	-	-	-	+	+
αCD32	-	-	-	+	-	+

Pre-OIT	+	-	+	+	+	+
Post-OIT	-	+	+	+	-	-
Tolerance	-	-	-	-	+	+
αCD32	-	-	-	+	-	+

Basophils from a healthy non-milk allergy donor was passively sensitized using one of the participants' sera at the beginning of the oral immunotherapy (OIT) (pre-OIT), post-OIT, natural outgrowth of cow's milk allergy (tolerance) with or without anti-CD32 antibody, then stimulated by αS1-casein. Basophil activation was analyzed using the percentages of high expression and mean fluorescence intensity (MFI) of CD203c [(A) and (C)], and CD63 [(B) and (D)]. This is the representative data from the 3 experiments.
