# Supplementary material for: Changes in passively‐sensitized basophil activation to αS1‐casein after oral immunotherapy
Source: Immun Inflamm Dis. 2020 Mar 3;8(2):188–97. doi: 10.1002/iid3.294 (PMC7212200; doi:10.1002/iid3.294)
Supplement: Supplementary file 2 — Supporting information [file IID3-8-188-s002.pdf]

Supplementary Table 1. Backgrounds of the sera used for the analysis of the suppressive effect of post-OIT sera.

No.	Tolerated amount of cow's milk (mL)	Total IgE (IU/mL)	αS1-Casein specific-IgE (%)	αS1-Casein specific-IgG4 (%)	Status
1	1	766	28.3	2.4	pre
	165	623	13.6	4.4	1y
2	1.9	518	13.1	6.1	pre
	114	739	5.7	82.3	1y
3	0.2	1164	20.7	4.9	pre
	200	659	6.3	21.2	2y
4	0.2	213	24.2	4.0	pre
	200	207	4.0	32.0	3y

The sera listed above were used for the experiments to confirm the suppressive effect of post-OIT sera on passively sensitized basophil activation. We matched the same individual's pre-OIT and post-OIT (1, 2, and 3 y) sera for the experiments.

Supplementary Table 2. Correlations between immunoglobulins and tolerated amount of cow's milk

	r	95% CI	*P*-value
Total IgE (IU/mL)	0.044	−0.13 to 0.21	0.62
Cow’s milk specific-IgE (kU_A/L)	−0.42	−0.55 to −0.27	<0.001
Casein specific-IgE (kU_A/L)	−0.44	−0.56 to −0.29	<0.001
α-Lactalbumin specific-IgE (kU_A/L)	−0.25	−0.042 to −0.071	0.0074
β-Lactoglobulin specific-IgE (kU_A/L)	−0.22	−0.38 to −0.31	0.018
αS1-Casein specific-IgE (%)	−0.46	−0.58 to −0.31	<0.001
αS1-Casein specific-IgG4 (%)	0.078	−0.093 to 0.25	0.37
αS1-Casein specific-IgG4/IgE	0.42	0.27 to 0.55	<0.001

Pearson’s product-moment correlation coefficient was calculated between immunoglobulins and tolerated amount of cow’s milk including all participants during oral immunotherapy.

Supplementary Table 3. Correlations between basophil activation and αS1-casein-specific immunoglobulins

		r	95% CI	*P*-value
CD63 (%)	IgE (%)	0.54	0.41 to 0.65	<0.001
	IgG4 (%)	0.18	0.008 to 0.34	0.04
	IgG4/IgE	−0.23	−0.39 to −0.062	0.0078
CD63 (MFI)	IgE (%)	0.65	0.54 to 0.74	<0.001
	IgG4 (%)	0.17	−0.0042 to 0.32	0.056
	IgG4/IgE	−0.27	−0.42 to −0.1	0.0019
CD203c (%)	IgE (%)	0.48	0.33 to 0.6	<0.001
	IgG4 (%)	0.18	0.0049 to 0.34	0.044
	IgG4/IgE	−0.21	−0.37 to −0.045	0.014
CD203c (MFI)	IgE (%)	0.58	0.45 to 0.68	<0.001
	IgG4 (%)	0.083	−0.88 to 0.25	0.34
	IgG4/IgE	−0.31	−0.46 to −0.15	<0.001

Pearson's product-moment correlation coefficient was calculated between basophil activation stimulated by αS1-casein and αS1-casein-specific immunoglobulins including all participants during oral immunotherapy.
MFI, mean fluorescence intensity

Supplementary Table 4. Correlations between basophil activation and tolerated amount of cow's milk

	r	95% CI	*P*-value
CD63 (%)	−0.51	−0.63 to −0.37	<0.001
CD63 (MFI)	−0.58	−0.68 to −0.46	<0.001
CD203c (%)	−0.45	−0.57 to −0.30	<0.001
CD203c (MFI)	−0.49	−0.61 to −0.35	<0.001

Pearson's product-moment correlation coefficient was calculated between basophil activation stimulated by αS1-casein and tolerated amount of cow's milk including all participants during oral immunotherapy.
MFI, mean fluorescence intensity
